# Green Synthesis of Hexagonal-like ZnO Nanoparticles Modified with Phytochemicals of Clove (*Syzygium aromaticum*) and *Thymus capitatus* Extracts: Enhanced Antibacterial, Antifungal, and Antioxidant Activities

**DOI:** 10.3390/ma17174340

**Published:** 2024-09-02

**Authors:** Kheira Haiouani, Sherif Hegazy, Huda Alsaeedi, Mikhael Bechelany, Ahmed Barhoum

**Affiliations:** 1 Department of Chemistry, Faculty of Exact Sciences and Informatics, Djelfa University, Djelfa 17000, Algeria; k.haiouani@univ-djelfa.dz; 2 Research Unit of Sustainable Chemistry, University of Oulu, P.O. Box 4300, FI-90014 Oulu, Finland; sherif.hegazy@oulu.fi; 3 Department of Chemistry, College of Science, King Saud University, Riyadh 11421, Saudi Arabia; halsaeedi@ksu.edu.sa; 4 Institut Européen des Membranes (IEM), UMR 5635, University of Montpellier, ENSCM, CNRS, F-34095 Montpellier, France; mikhael.bechelany@umontpellier.fr; 5 Functional Materials Group, Gulf University for Science and Technology (GUST), Masjid Al Aqsa Street, Mubarak Al-Abdullah 32093, Kuwait; 6 NanoStruc Research Group, Chemistry Department, Faculty of Science, Helwan University, Cairo 11795, Egypt

**Keywords:** green synthesis, zinc oxide nanoparticles, clove extract, *Thymus capitatus*, antioxidant activity, antibacterial activity, biocompatibility, eco-friendly synthesis

## Abstract

The green synthesis of ZnO NPs is becoming increasingly valued for its cost-effectiveness and environmental benefits. This study successfully synthesized hexagonal ZnO NPs using a combination of clove (*Syzygium aromaticum*) and *Thymus capitatus* extracts. The use of both extracts significantly improved the antibacterial and antioxidant properties of the ZnO NPs. By optimizing synthesis conditions, including ZnCl_2_ and extract concentrations, hexagonal wurtzite ZnO NPs were produced at room temperature with only drying at 80 °C without high-temperature annealing. The synthesized ZnO NPs exhibited a hexagonal morphology with an average particle size of 160 nm and a crystallite size of 30 nm. Energy-dispersive X-ray spectroscopy (SEM-EDX) confirmed the elemental composition of the ZnO NPs, showing a high carbon content (63.9 wt.%), reflecting the presence of phytochemicals from the extracts coated the ZnO NPs surface. The UV–Vis spectrum revealed an absorption peak at 370 nm and a bandgap energy of 2.8 eV due to lattice defects caused by organic impurities. The ZnO NPs demonstrated exceptional antioxidant activity, with a DPPH radical scavenging rate of 95.2%. They also exhibited strong antibacterial activity against both Gram-positive and Gram-negative bacteria, with inhibition zones of 25 mm against *Bacillus subtilis*, 26 mm against *Escherichia coli*, 24 mm against *Salmonella typhimurium*, 22 mm against *Klebsiella pneumoniae*, 21 mm against *Staphylococcus aureus*, 20 mm against *Staphylococcus hominis*, and 18 mm against *Bacillus subtilis* at 200 ppm. Furthermore, significant antifungal activity was observed against *Candida albicans*, with an inhibition zone of 35 mm at the same concentration. These findings underscore the effectiveness of using combined plant extracts for producing ZnO NPs with controlled morphology and enhanced biological properties, highlighting their potential for various biomedical applications.

## 1. Introduction

Zinc oxide nanoparticles (ZnO NPs) have garnered considerable attention due to their unique characteristics, making them valuable across diverse fields such as optics, electronics, food packaging, and medicine [1,2,3,4]. Their exceptional physicochemical properties enable their use in various applications, including antibacterial agents, catalysts, and electronic devices [5,6]. However, traditional methods for producing ZnO NPs often involve toxic chemicals and energy-intensive processes, posing significant environmental and health hazards [7,8]. Consequently, there is a growing interest in green synthesis techniques that use plant extracts, providing a more sustainable and eco-friendlier alternative [9]. These green methods not only minimize the harmful environmental impacts associated with conventional synthesis but also offer the potential for producing biocompatible nanoparticles, aligning with the principles of green chemistry [10]. The shift toward green synthesis emphasizes the importance of morphology control in ZnO NPs, allowing for tailored properties that enhance their performance in specific applications without compromising efficiency and functionality.

Natural products such as citronella, orange, thyme, lavender, grapefruit, bergamot, cinnamon, and rosemary essential oils exhibit antimicrobial, antioxidant, and antifungal activities, making them ideal for use in innovative antibacterial and antifungal therapies [11]. These essential oils, combined with plant extracts derived from various plant parts like leaves, fruits, seeds, roots, and whole plants, are abundant in phytochemicals such as phenolic compounds, alkaloids, flavonoids, and terpenoids [12,13,14,15,16,17,18,19,20]. These bioactive compounds serve as natural reducing and stabilizing agents in the green synthesis of ZnO NPs, aiding in the formation of nanoparticles with controllable size, shape, and crystallinity [21]. The ability to precisely control these parameters is crucial for optimizing the physical and chemical properties of ZnO NPs, which significantly impacts their biological functionality and potential applications [22]. Fine-tuning the synthesis process allows researchers to develop ZnO NPs with specific attributes, enhancing their effectiveness in targeted applications. This control over nanoparticle morphology is vital for maximizing their performance, particularly in the biomedical field, where precise specifications are often required. The green synthesis approach not only facilitates the environmentally friendly production of ZnO NPs but also ensures that the NPs meet the desired criteria for specific uses.

Green-synthesized ZnO NPs have demonstrated a wide range of biomedical applications, including notable antibacterial, anti-inflammatory, and potential anticancer properties [23,24,25,26,27]. These NPs have been successfully integrated into wound dressings, drug delivery systems, and biosensing and imaging technologies [23,28]. Compared with traditional synthesis methods, green synthesis techniques offer numerous advantages, such as improved biocompatibility, lower toxicity, and enhanced environmental sustainability [29,30,31]. These benefits make green-synthesized ZnO NPs highly attractive for various biomedical uses, where safety and effectiveness are paramount [32]. The increasing demand for sustainable and biocompatible materials in healthcare further indicates the importance of developing green synthesis methods that align with these requirements, positioning ZnO NPs as a promising candidate for future biomedical innovations. Control over morphology and size in green synthesis is pivotal in achieving these advantages, as it directly influences the NPs’ interaction with biological systems and their ultimate efficacy in medical applications.

The aim of this study is to establish a novel green synthesis approach for producing ZnO nanoparticles (NPs) by utilizing phytochemical-rich extracts from clove (*Syzygium aromaticum*) and *Thymus capitatus*. This method involves synthesizing crystalline ZnO NPs at room temperature (25 °C) with minimal alkali and simple drying at 80 °C, thus avoiding the need for high-temperature annealing. We use hydrodistillation extracts from clove, known for its eugenol, and *Thymus capitatus*, which is rich in phenolic compounds and essential oils. The novelty of this approach lies in using these combined plant extracts as natural reducing and capping agents, which stabilize the nanoparticles and enhance their properties. This method not only allows for precise control over the size and shape of the ZnO NPs but also prevents their agglomeration and significantly improves their antibacterial and antioxidant activities. The advantages of this green synthesis method include its environmental friendliness, cost-effectiveness, and efficiency, offering a sustainable alternative to traditional high-temperature synthesis techniques. By focusing on particle morphology and optimizing phytochemical use, this research aims to produce ZnO NPs with enhanced functionality for various applications.

## 2. Materials and Methods

### 2.1. Materials and Reagents

All chemicals were procured from Sigma-Aldrich (St. Louis, MO, USA) and were of analytical grade, ensuring the purity and reliability needed for accurate experimental results. The main chemicals used include zinc chloride (ZnCl_2_, MW: 136.30 g/mol, purity ≥ 98%), which serves as a zinc source for the synthesis of ZnO NPs. Sodium hydroxide (NaOH, MW: 40.00 g/mol, purity ≥ 98%) was used to adjust the pH during nanoparticle synthesis to facilitate the precipitation of ZnO NPs. 2,2-Diphenyl-1-picrylhydrazyl (DPPH, C_18_H_12_N_5_O_6_, MW: 394.32 g/mol) was employed in the antioxidant assay to assess the free radical scavenging capability of the synthesized NPs. Ethanol (C_2_H_5_OH, purity ≥ 99.8%) was used as a solvent for DPPH. Deionized water was used throughout the experiments for all aqueous solutions and cleaning purposes. Plant materials include clove (*Syzygium aromaticum*) dried buds and *Thymus capitatus* aerial parts, both sourced locally for extraction. All reagents were stored according to manufacturer instructions to maintain stability and integrity.

### 2.2. Preparation of Plant Extracts

The preparation of clove and *Thymus capitatus* extracts was conducted via hydrodistillation, a method chosen for its ability to effectively isolate essential oils and bioactive compounds [33]. Clove (*Syzygium aromaticum*) dried flower buds were purchased from a local market. Initially, the buds were rinsed three times with distilled water to remove any surface impurities and disinfected with a 5wt% sodium hypochlorite (NaOCl) solution for 10 min to eliminate microbial contamination. After disinfection, the buds were thoroughly rinsed with distilled water and allowed to dry at room temperature. Once dried, the clove buds were ground into a fine powder using a mechanical mortar and pestle. *Thymus capitatus* aerial parts were harvested from Tiaret, Algeria. The samples were air-dried in a shaded area to prevent loss of volatile compounds and stored in sealed containers at ambient temperature until extraction. For the hydrodistillation process, 200 g of *Thymus capitatus* powder was placed in a 1000 mL round-bottom flask with 500 mL of deionized water and subjected to hydrodistillation for 3 h in a Deryng apparatus [33]. The measurements of the distillation time began after the first drop of distillate appeared. The extract was then stored in a sealed container at 4 °C for further experiments. Clove extract was prepared using the same procedure described for *Thymus capitatus*, ensuring uniformity in extraction conditions for both plants. The extracts were labeled and stored under refrigeration to maintain their chemical stability and bioactivity.

### 2.3. Synthesis of Zinc Oxide Nanoparticles

To optimize the synthesis of ZnO NPs, various experimental conditions were tested to assess their impact on antioxidant and antibacterial activities, particularly focusing on ZnCl_2_ concentration and plant extract volumes (refer to Table 1). The synthesis employed a green chemistry approach, with plant extracts acting as caping (shape control) agents. ZnCl_2_ was dissolved in 50 mL of deionized water under magnetic stirring at 25 °C to ensure complete dissolution, and the prepared clove and Thymus capitatus extracts were added dropwise while maintaining constant stirring. After 30 min of interaction between the ZnCl_2_ and the bioactive compounds in the extracts, the pH of the reaction mixture was adjusted to approximately 8 by the dropwise addition of 0.1 M NaOH, under vigorous stirring, to promote nucleation and growth of ZnO NPs. The mixture was then subjected to 30 min of ultrasonication to enhance dispersion and homogeneity. After allowing the reaction mixture to settle for 24 h, the ZnO NPs were collected via centrifugation at 10,000 rpm for 15 min, followed by multiple washes with deionized water and ethanol to eliminate unreacted precursors and impurities. The purified nanoparticles were dried at 80 °C to form a fine powder, which was then stored in a desiccator for subsequent characterization and biological activity evaluations.

### 2.4. Characterization of ZnO Powder Particles

To effectively characterize the synthesized ZnO NPs, various analytical techniques are employed to determine their morphological and structural properties. UV–Visible Spectral Analysis is conducted using a Cary-8454 spectrophotometer (Agilent, Santa Clara, CA, USA), scanning a wavelength range from 200 to 800 nm. For this analysis, prepare a colloidal suspension of ZnO NPs in deionized water at a concentration of 0.1 mg/mL. Place 1–2 mL of this suspension in a quartz cuvette and perform the scan. This technique helps identify the characteristic absorption peak of ZnO NPs, typically around 370 nm, which corresponds to the band-gap transition.

Scanning Electron Microscopy (SEM) and Energy-Dispersive X-ray Spectroscopy (EDX) are used to examine the surface morphology, size distribution, and elemental composition of ZnO NPs. For SEM, drop-cast a small volume (~10 µL) of the NP suspension onto a silicon wafer or carbon-coated SEM stub and allow it to dry. The samples were coated with gold sputtering. Use a Hitachi SUM800 microscope operating at 20 kV to capture images at various magnifications. EDX analysis, performed in conjunction with SEM, helps identify the elemental composition by detecting peaks for zinc (Zn) and oxygen (O), confirming the presence of ZnO.

X-ray Diffraction (XRD) is employed to investigate the crystallographic structure of ZnO NPs. Prepare a fine powder of the dried ZnO NPs by drying and grinding the suspension. Place approximately 1–2 g of this powder into an XRD sample holder. Use a Philips PW1730 diffractometer with Cu-Kα radiation (λ = 1.5406 Å) to scan over a 2θ range from 5° to 80°, with a step size of 0.02° and a scan rate of 2°/min. The XRD pattern obtained reveals the phase purity and crystallite size of ZnO NPs. Analyze the diffraction peaks to confirm the ZnO phase and calculate the crystallite size using the Scherrer equation based on the full width at half maximum (FWHM) of the prominent peaks.

### 2.5. Antioxidant Activity Assay

The antioxidant activity of the synthesized ZnO NPs was evaluated using a modified version of the 2,2-Diphenyl-1-Picrylhydrazyl (DPPH) assay, a widely used method to assess free radical scavenging capabilities. A fresh solution of DPPH (0.2 mM) was prepared in ethanol. Different concentrations of ZnO NPs (220, 500, 750, and 1000 μg/mL) were prepared by dispersing the NPs in ethanol. In each assay, 1 mL of DPPH solution was mixed with 1 mL of the ZnO NPs dispersion and incubated in the dark at room temperature for 30 min. Ascorbic acid (Vitamin C) was used as a standard antioxidant for comparison [34]. A concentrated solution of ascorbic acid was prepared in ethanol at a concentration of 1 mg/mL. From this stock solution, different concentrations (220, 500, 750, and 1000 μg/mL) were prepared by dilution. In each assay, 1 mL of DPPH solution was mixed with 1 mL of the ascorbic acid solution and incubated in the dark at room temperature for 30 min.

The absorbance of the reaction mixtures was measured at 517 nm using a UV–Vis spectrophotometer. The absorbance at 517 nm is measured in the DPPH assay because it corresponds to the maximum absorbance wavelength of the DPPH radical in its unreacted form. DPPH is a stable free radical with a characteristic deep violet color, and it absorbs strongly at 517 nm. When an antioxidant, such as ZnO NPs or ascorbic acid, scavenges the DPPH radicals, they are reduced, resulting in a color change from violet to yellow. This decrease in absorbance at 517 nm is directly proportional to the free radical scavenging activity of the antioxidant, making it an ideal measurement point for evaluating antioxidant activity [35]. The percentage of DPPH radical scavenging activity was calculated using the formula (Equation (1)):

(1) $$\mathrm{Scavenging}\;\mathrm{Activity}\;(\%)=\frac{A_{O}-A_{S}\;}{A_{O}\;}\times 100$$

$A_{O}$ is the absorbance of the DPPH solution without NPs, and $A_{S}$ is the absorbance in the presence of ZnO NPs. This assay provided insights into the potential use of ZnO NPs as antioxidant agents, with higher scavenging activity indicating greater efficacy.

### 2.6. Antibacterial Activity Assay

The antibacterial activity of the synthesized ZnO NPs was assessed using the disc diffusion method against a panel of bacterial strains, including both Gram-positive and Gram-negative species. The bacterial strains used were *Staphylococcus aureus* (ATCC 25932), *Staphylococcus hominis* (ATCC 27844), *Bacillus subtilis* (ATCC 25973), *Escherichia coli* (ATCC 25922), *Salmonella typhimurium* (ATCC 14028), and *Klebsiella pneumoniae* (ATCC 13883). These strains were maintained on nutrient agar slants at 4 °C until use. A 24 h old culture of each bacterium was prepared by inoculating the strains in nutrient broth and incubating at 37 °C. The bacterial suspension was adjusted to a 0.5 McFarland standard (approximately 10^6^ CFU/mL, i.e. colony-forming units) using sterile physiological saline. Muller Hinton Agar (MHA) plates were prepared, and the surface was inoculated with the standardized bacterial suspension using a sterile cotton swab. Sterile paper discs (6 mm in diameter) were loaded with 20 μL of ZnO NPs suspensions at concentrations of 20, 50, 100, and 200 μg/disc. The discs were placed equidistantly on the inoculated MHA plates, with three replicates per concentration. Ciprofloxacin (5 μg/disc) served as a positive control, and sterile deionized water was used as a negative control. The plates were incubated at 37 °C for 24 h, after which the zones of inhibition (ZOI) around each disc were measured in millimeters using a digital caliper. The antibacterial efficacy of ZnO NPs was evaluated based on the size of the ZOI, with larger zones indicating higher antibacterial activity.

### 2.7. Statistical Analysis

Statistical analysis was performed to assess result significance and reliability. Antioxidant and antimicrobial data were gathered from multiple independent experiments, each conducted in triplicate, to ensure robustness and reproducibility. Results are presented as mean ± standard deviation.

## 3. Results and Discussions

### 3.1. Green Synthesis of Hexagonal ZnO NPs

In the conventional synthesis of ZnO NPs, the process begins with a precipitation reaction between ZnCl_2_ and NaOH, resulting in the formation of a white precipitate, Zn(OH)_2_. This precipitate serves as an intermediate that must undergo further processing to convert into ZnO NPs. The transformation of Zn(OH)_2_ into ZnO involves a thermal decomposition reaction. However, drying at temperatures around 80 °C primarily removes water and does not provide sufficient energy to fully decompose Zn(OH)_2_ into ZnO, leading to incomplete nanoparticle formation during the drying phase alone. Annealing at higher temperatures is required to achieve the complete conversion of Zn(OH)_2_ to ZnO, facilitating particle growth and enhancing crystallinity. Conventional methods typically involve heating at temperatures ranging from 400 °C to 800 °C to optimize nanoparticle size and morphology. Studies, such as those by Jyoti et al. [36], demonstrate how varying reaction parameters like temperature and NaOH concentration affect ZnO NPs size and morphology. For example, increasing temperatures from 800 °C to 1000 °C and adjusting NaOH concentrations from 2 M to 10 M resulted in particle sizes ranging from 20 nm to 350 nm and morphologies shifting from spherical to rod-like and polygonal shapes. These methods, while effective, require significant energy and extended processing times, highlighting the critical role of annealing in optimizing ZnO NPs properties.

This study introduces a novel green synthesis method for producing ZnO NPs using hydrodistilled extracts from clove (*Syzygium aromaticum*) and *Thymus capitatus*. Hydrodistillation offers significant benefits by preserving volatile compounds that are sensitive to heat and oxidation, using gentle heat and steam instead of harsh solvents [33]. This approach ensures high-quality extracts with intact aromatic and bioactive substances, resulting in high-purity extracts with minimal impurities and enhanced environmental sustainability. In addition, this method facilitates the formation of ZnO NPs with minimal alkali and avoids extensive thermal processing. The phenolic compounds in clove and *Thymus capitatus* extracts act as natural stabilizing agents, guiding the formation of well-defined hexagonal ZnO NPs with a wurtzite structure under mild alkaline conditions (pH 8) and low drying temperatures of 80 °C [36,37,38,39,40]. By eliminating the need for high-temperature annealing and synthetic surfactants, this approach simplifies the synthesis process, reduces energy consumption, and enhances environmental sustainability. The ZnO NPs produced, coated with phytochemicals, exhibit improved surface area, reactivity, and enhanced biological activities, including antioxidant and antibacterial effects, making them highly suitable for various biomedical applications.

Rajae Zahli et al. [41] performed a quantitative analysis of essential oils from Thymus capitatus and Syzygium aromaticum using GC-MS spectroscopy. For Thymus capitatus, the essential oil is primarily composed of carvacrol (73.52%), which facilitates the formation of ZnO NPs and stabilizes them through its antioxidant properties, thus controlling nanoparticle morphology and preventing aggregation. Other components like o-cymene (7.72%) and borneol (5.06%) aid in modifying the chemical environment and stabilizing intermediate phases during synthesis. Minor constituents, including trans-caryophyllene (4.88%), linoelaidic acid (3.58%), linalool (3.13%), β-myrcene (1.06%), and γ-terpinene (1.05%), also contribute to the growth and stability of ZnO NPs. In clove extract, eugenol (82.06%) acts as a crucial stabilizing agent, preventing agglomeration and ensuring well-defined ZnO nanoparticle formation. β-Caryophyllene (14.52%) may function as a capping agent to guide nanoparticle growth, while other constituents like caryophyllene oxide (0.82%), eugenyl acetate (0.83%), and humulene (1.77%) enhance the nanoparticles’ antimicrobial and antioxidant activities. This diverse composition supports the efficient and controlled synthesis of ZnO nanoparticles with desirable properties.

### 3.2. Optimization of the Synthesis Process

To optimize the synthesis of ZnO NPs, the antioxidant and antibacterial activities were under various experimental conditions, focusing on the impact of ZnCl_2_ concentration and plant extract volumes (refer to Table 1).

The antioxidant capability measured using the DPPH assay identified Experiment 6 as the most effective, with a DPPH inhibition of 95.2%. This optimal performance results from the balanced use of 2.0 g ZnCl_2_ and 10 mL each of clove and *Thymus capitatus* extracts. This combination enhances nanoparticle stabilization and dispersion, resulting in superior radical scavenging efficiency. In contrast, Experiments 1 and 2—both utilizing 3.0 g ZnCl_2_ but with different extract volumes (15 mL clove and 5 mL *Thymus capitatus* for Experiment 1, and 5 mL clove and 15 mL *Thymus capitatus* for Experiment 2)—showed lower antioxidant activities of 85.5% and 86.3%, respectively. The imbalance in extract volumes likely led to inefficient stabilization of the nanoparticles, causing aggregation and reduced antioxidant efficacy. Experiment 7, which used 4.0 g ZnCl_2_ and 5 mL each of clove and *Thymus capitatus* extracts, demonstrated an antioxidant activity of 93.3%. This is lower than Experiment 6, indicating that while the increased ZnCl_2_ concentration improved some properties, it did not enhance antioxidant activity as effectively as the lower concentration used in Experiment 6.

Experiment 6 also excelled in antibacterial activity, showing ZOI of 18 mm against *Klebsiella pneumoniae* and 25 mm against *Bacillus subtilis*. This indicates that the lower ZnCl_2_ concentration (2.0 g) and balanced extract volumes positively influence the size and morphology of ZnO NPs, leading to enhanced bacterial inhibition. In comparison, Experiment 1 showed ZOI of 14 mm against *Klebsiella pneumoniae* and 10 mm against *Bacillus subtilis*, while Experiment 2 had ZOI of 12 mm and 15 mm, respectively. The differing extract volume ratios in these experiments affected nanoparticle size and stability, with excess extracts leading to inefficient capping and stabilization. Experiment 7, which had a ZnCl_2_ concentration of 4.0 g and unequal extract volumes (5 mL each), showed ZOI of 9 mm against *Klebsiella pneumoniae* and 10 mm against *Bacillus subtilis*. The reduced antibacterial activity in Experiment 7, despite the higher ZnCl_2_ concentration, suggests that the imbalance in extract volumes and high ZnCl_2_ concentration compromised nanoparticle stability and effectiveness.

Experiments 3, 4, and 5, with varying ZnCl_2_ concentrations and extract volumes, showed intermediate results. Experiment 3, using 3.0 g ZnCl_2_ and 10 mL each of clove and *Thymus capitatus* extracts, achieved a DPPH inhibition of 91.2% and moderate antibacterial activity with ZOI of 15 mm and 16 mm. Experiment 4, with a higher ZnCl_2_ concentration of 4.0 g and balanced extracts, demonstrated a DPPH inhibition of 92.0% and ZOI of 14 mm and 15 mm. These results indicate that while higher ZnCl_2_ concentrations can improve certain properties, they can also lead to suboptimal stabilization if not combined with appropriate extract volumes. Experiment 5, with 5.5 g ZnCl_2_ and balanced extract volumes, achieved a DPPH inhibition of 93.4% but showed lower antibacterial efficacy with ZOI of 11 mm and 12 mm, likely due to excess ZnCl_2_ affecting nanoparticle stabilization.

The results highlight the importance of optimizing ZnCl_2_ concentration and plant extract volumes. Experiment 6, with a lower ZnCl_2_ concentration and equal volumes of clove and *Thymus capitatus* extracts, emerged as the best combination, yielding ZnO NPs with exceptional antioxidant and antibacterial properties. Detailed characterization of Experiment 6, using SEM, XRD, SEM-EDX, and UV–Vis spectrophotometry, confirmed the nanoparticles’ optimal size, shape, and stability, demonstrating their potential for various applications.

### 3.3. Morphological and Elemental Analysis

**Figure 1:**
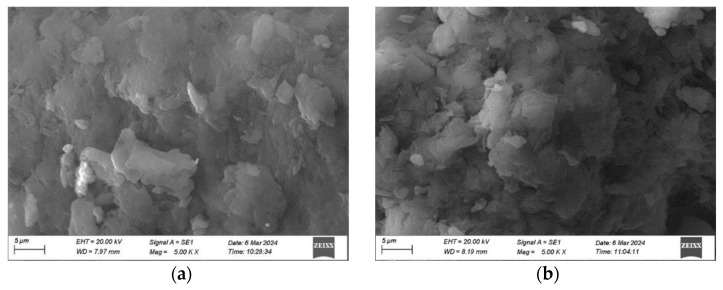
SEM images of ZnO nanoparticles synthesized under varying conditions: (**a**) Irregularly aggregated ZnO sub-microparticles from Experiment 1, prepared with 3.0 g ZnCl_2_, 15 mL clove extract, and 5 mL *Thymus capitatus* extract, showing particle sizes exceeding 500 nm. (**b**) Irregularly aggregated ZnO sub-microparticles from Experiment 2, prepared with 3.0 g ZnCl_2_, 5 mL clove extract, and 15 mL *Thymus capitatus* extract, also exhibiting sizes greater than 500 nm.

**Figure 2:**
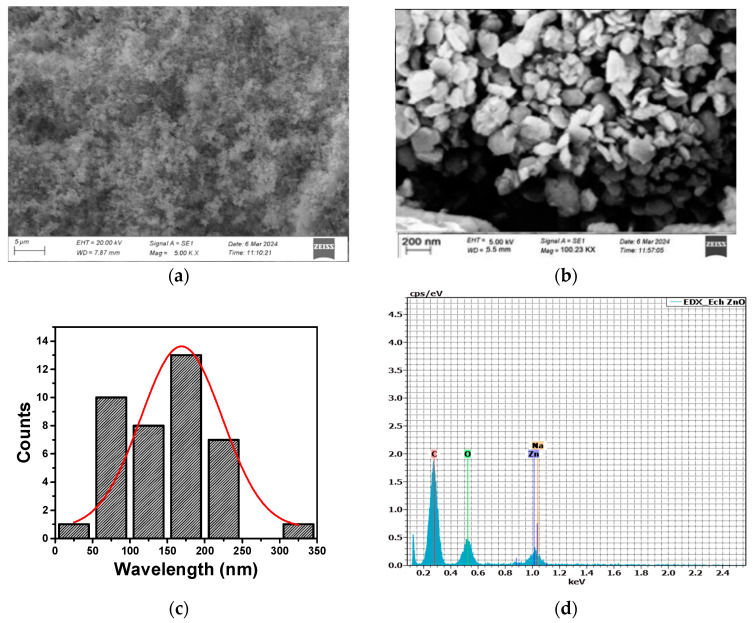
SEM analysis of ZnO NPs synthesized under optimized conditions: (**a**) Hexagonal ZnO NPs with an average particle size of approximately 160 nm from Experiment 6, using 2.0 g ZnCl_2_, 10 mL clove extract, and 10 mL *Thymus capitatus* extract. (**b**) Additional SEM image showing the uniform hexagonal morphology. (**c**) Histogram depicting particle size distribution estimated from SEM images using ImageJ Software. (**d**) EDX spectrum showing elemental composition: carbon (C), oxygen (O), zinc (Zn), and sodium (Na).

SEM analysis highlights the substantial influence of ZnCl_2_ concentration and plant extract volumes on the size and morphology of ZnO NPs. In Experiment 1, 3.0 g of ZnCl_2_ combined with uneven extract volumes (15 mL clove and 5 mL *Thymus capitatus*) resulted in sub-microparticles with irregular, aggregated morphologies and sizes exceeding 500 nm (Figure 1a). The high ZnCl_2_ concentration accelerated nucleation and growth, but without sufficient stabilization from the plant extracts, this led to uncontrolled particle aggregation. The imbalance in extract volumes further exacerbated this issue, with excessive clove extract failing to control the nanoparticles uniformly. Conversely, Experiment 2, using the same ZnCl_2_ concentration but reversed extract volumes (5 mL clove and 15 mL *Thymus capitatus*), also produced irregular, aggregated particles over 500 nm in size (Figure 1b). Here, inadequate distribution of phytochemicals led to poor capping and stabilization. Experiment 6, with a reduced ZnCl_2_ concentration of 2.0 g and balanced extract volumes (10 mL each), achieved well-defined hexagonal-shaped ZnO NPs with an average size of approximately 160 nm (Figure 2a,b). This optimized condition facilitated controlled nucleation and growth, with phytochemicals from clove (eugenol, caryophyllene, and acetyl eugenol) and *Thymus capitatus* (thymol, carvacrol, and p-cymene) effectively capping and stabilizing the ZnO NPs. These results are in agreement with previous studies [41,42,43]. Velsankar et al. [37] synthesized hexagonal-shaped ZnO NPs using *Echinochloa frumentacea* grain powder extract. Chaudhary et al. [44] synthesized ZnO NPs using aqueous Aloe vera peel extract. Their findings confirmed the production of crystalline ZnO NPs, with sizes ranging from 50 to 220 nm and a hexagonal shape.

During the synthesis process, the phytochemicals from clove and *Thymus capitatus* extracts selectively adhere to the surface of the ZnO NPs. However, the specific compounds that bind to the ZnO NP surface and any alterations in their chemical structures due to reactions with ZnCl_2_ and NaOH remain unclear. SEM-EDX analysis was employed to verify the presence of these phytochemicals and examine their interactions with the ZnO NPs. EDX results (Figure 2) indicate the elemental composition of the ZnO NPs as follows: carbon (C) at 63.9 ± 17.9 wt.% (76.0 ± 17.9 at.%), oxygen (O) at 23.8 ± 8.7 wt.% (21.2 ± 8.7 at.%), zinc (Zn) at 12.1 ± 1.7 wt.% (2.6 ± 1.7 at.%), and sodium (Na) at 0.2 ± 0.1 wt.% (0.1 ± 0.1 at.%). The high carbon content reflects the deposition of phytochemicals on the ZnO NP surface, while the minimal sodium content, resulting from NaOH used for pH adjustment, has a negligible impact on the ZnO NPs’ overall properties. NaOH is used in the synthesis process to adjust the pH and provide a basic environment, which is crucial for the formation of ZnO NPs.

### 3.4. XRD Analysis of Hexagonal ZnO NPs

**Figure 3:**
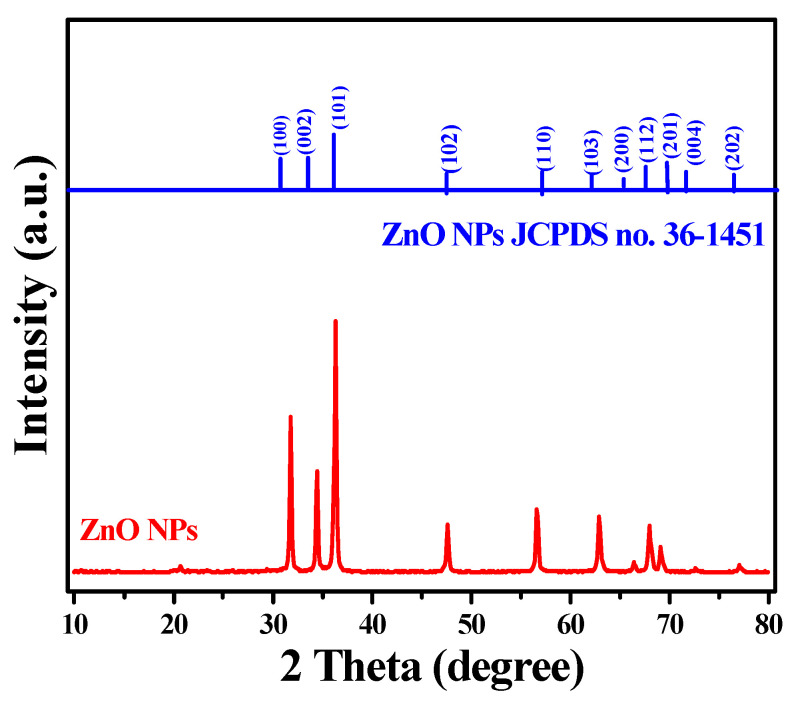
XRD diffraction pattern of the hexagonal ZnO NPs synthesized from conditions in Experiment 6 (Table 1).

XRD analysis of the synthesized ZnO NPs reveals distinct diffraction peaks at 2θ values of approximately 31.8°, 34.4°, and 36.2°, corresponding to the (100), (002), and (101) crystal planes of the hexagonal wurtzite structure (Figure 3). These diffraction patterns match the standard ZnO reference (JCPDS card no. 36-1451), confirming the formation of a hexagonal wurtzite crystal system. The crystallite size, calculated using the Debye–Scherrer equation, is approximately 30 nm, indicative of well-defined nanocrystals. However, the discrepancy between this crystallite size and the larger particle size observed via scanning electron microscopy (SEM) (~160 nm) suggests that the nanoparticles are polycrystalline, with each particle consisting of multiple crystallites, averaging about five. The XRD patterns may be influenced by the phytochemicals from the plant extracts used during synthesis. Phytochemicals such as flavonoids, phenolics, and terpenoids, acting as capping agents, may interact with the ZnO crystal surfaces, potentially affecting crystal growth and orientation. These compounds might bind to specific crystal facets, altering growth kinetics along different crystallographic directions, resulting in slight peak broadening or reduced peak intensity. Despite these interactions, the XRD data predominantly indicate a well-crystallized hexagonal wurtzite structure, with minimal disruption to the crystal lattice from the phytochemicals.

### 3.5. UV–Vis Analysis of Hexagonal ZnO NPs

**Figure 4:**
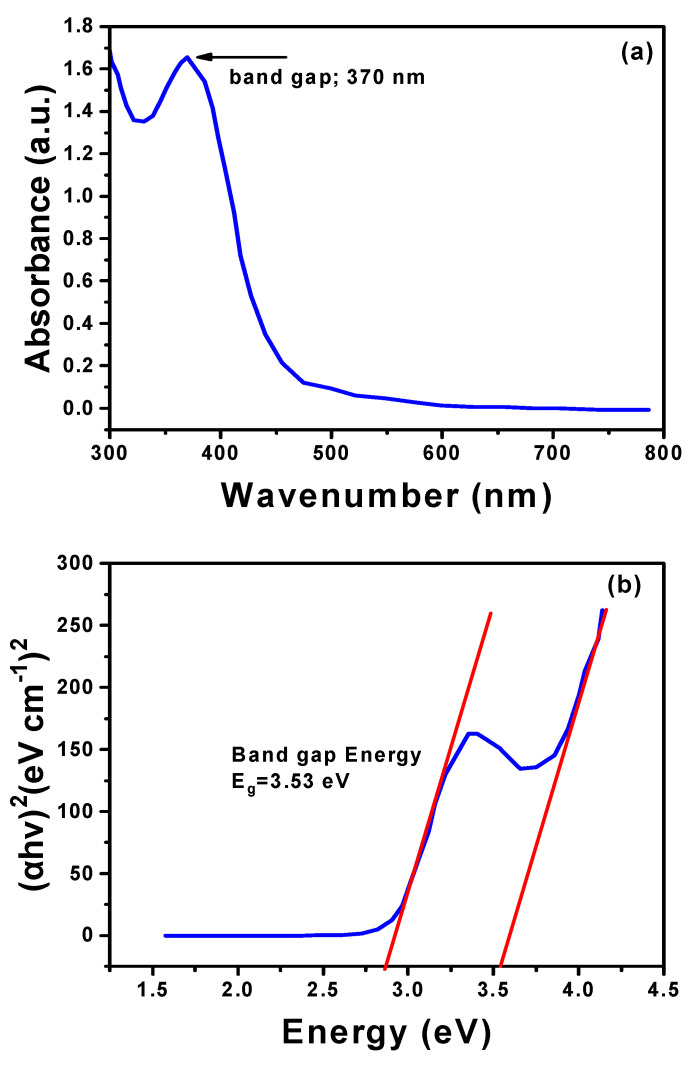
UV–Vis analysis of hexagonal ZnO NPs synthesized under Experiment 6 conditions (Table 1). (**a**) UV–Vis absorption spectrum. (**b**) Band gap energy diagram derived from the UV–Vis data.

The UV–Vis spectrum of the synthesized hexagonal ZnO NPs, as illustrated in Figure 4a, displays a prominent absorption peak at 370 nm, which is characteristic of ZnO’s bandgap transitions, confirming the successful formation of the nanoparticles. To more accurately estimate the bandgap energy, the Tauc plot method was employed, as shown in Figure 4b. This technique involved plotting (αhν)^2^ against photon energy (hν), where the absorption coefficient (α) is derived from the UV–Vis data. A tangent drawn along the absorption edge of the UV–Vis spectrum allowed the calculation of a bandgap energy of approximately 2.8 eV. Although this value is lower than the conventional bandgap of bulk ZnO (around 3.10 eV to 3.37 eV), it aligns with the properties of ZnO nanostructures, potentially due to quantum confinement effects or surface defects [40,41]. The reduction in bandgap energy is further supported by the extended absorption tail observed in the UV–Vis spectrum, which stretches beyond 500 nm into the visible light region. Phytochemicals likely contributed to this shift by interacting with the ZnO surfaces, thereby influencing their electronic properties. This extended absorption and reduced bandgap highlight the ZnO nanoparticles’ potential for enhanced optoelectronic and photocatalytic applications, as the nanoparticles can absorb a broader range of light wavelengths, improving their efficiency in such technologies [45].

### 3.6. Antioxidant Activity of Hexagonal ZnO NPs

**Figure 5:**
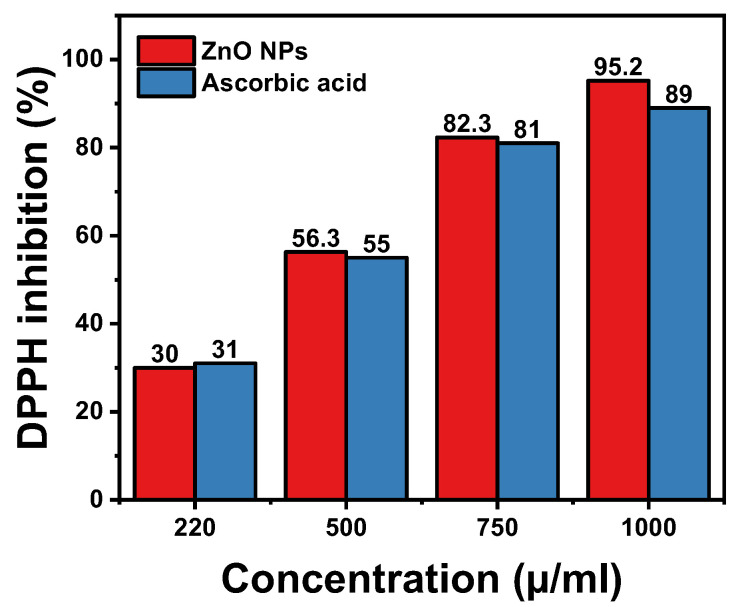
Antioxidant activity of hexagonal wurtzite ZnO NPs and Ascorbic acid, showing % DPPH inhibition at various concentrations.

Antioxidant activity of hexagonal ZnO NPs synthesized using combined plant extracts of clove (*Syzygium aromaticum*) and *Thymus capitatus* was assessed using the DPPH radical scavenging assay (Figure 5 and Table 2). In this assay, the ability of a substance to donate hydrogen atoms and neutralize DPPH radicals is measured. *Ascorbic acid*, a well-established antioxidant, was used as a reference standard. The results showed a clear dose-dependent increase in % DPPH inhibition for both the ZnO NPs and *Ascorbic acid*. At a concentration of 1000 µg/mL, the hexagonal ZnO NPs achieved an impressive % DPPH inhibition of 95.2%, which was higher than the 89.0% inhibition observed for Ascorbic acid at the same concentration. This indicates the remarkable antioxidant potential of the synthesized ZnO NPs. Additionally, the IC50 values, which represent the concentration required for 50% DPPH inhibition, were 434.06 µg/mL for ZnO NPs and 442.96 µg/mL for Ascorbic acid (Table 2). The slightly lower IC50 value for ZnO NPs suggests a higher efficiency in scavenging DPPH radicals, reinforcing their strong antioxidant capabilities.

The enhanced antioxidant activity of the ZnO NPs can be attributed to several factors. Phytochemicals from clove and *Thymus capitatus* extracts are known for their potent antioxidant properties. These compounds act as capping (shape control) agents during the synthesis of ZnO NPs and contribute to the radical scavenging capabilities of the nanoparticles. Furthermore, the hexagonal structure of the ZnO NPs provides a high surface area, increasing the number of reactive sites available for antioxidant reactions. This larger surface area facilitates more effective interaction with free radicals, thereby enhancing the nanoparticles’ ability to neutralize them. Collectively, these factors contribute to the superior antioxidant performance of the ZnO NPs, highlighting their potential for applications requiring robust antioxidant properties.

### 3.7. Antibacterial Activity of Hexagonal ZnO NPs

**Figure 6:**
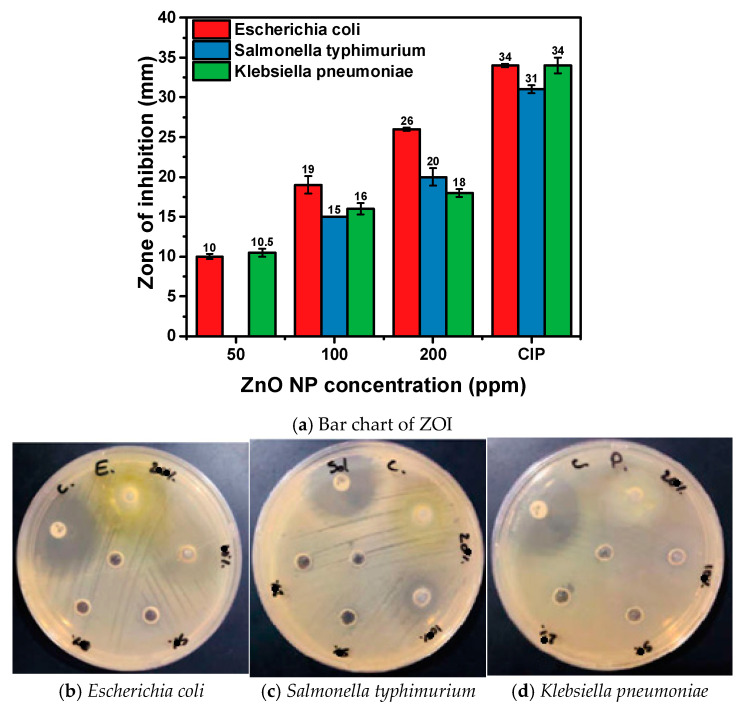
Evaluation of Antibacterial Activity of Hexagonal Wurtzite ZnO NPs Against Gram-negative bacteria. (**a**) Bar chart illustrating the ZOI observed for various concentrations of ZnO NPs. Camera image showing ZOI around the ZnO NP-treated areas for (**b**) *Escherichia coli ATCC 25922*. (**c**) ZOI for *Salmonella typhimurium ATCC 14028*. (**d**) ZOI for *Klebsiella pneumoniae ATCC 13883*.

**Figure 7:**
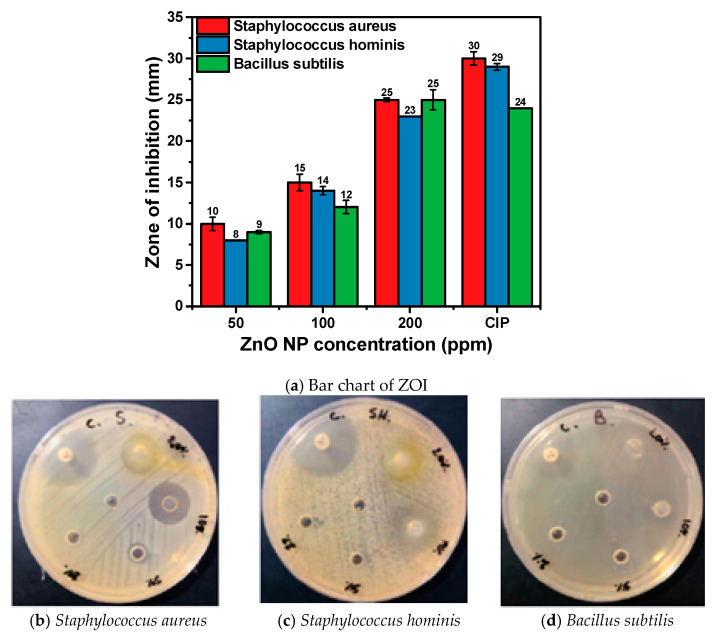
Evaluation of Antibacterial Activity of Hexagonal Wurtzite ZnO NPs Against Gram-positive bacteria. (**a**) Bar chart illustrating the ZOI observed for various concentrations of ZnO NPs. Camera image showing ZOI around the ZnO NP-treated areas for (**b**) *Staphylococcus aureus* ATCC 25932. (**c**) ZOI for *Staphylococcus hominis* ATCC 27844. (**d**) ZOI for *Bacillus subtilis* ATCC 25973.

The antibacterial efficacy of ZnO NPs synthesized using combined clove (*Syzygium aromaticum*) and *Thymus capitatus* extracts was assessed against a range of bacterial strains, including both Gram-negative and Gram-positive bacteria. Various concentrations of ZnO NPs (200, 100, 50, and 20 ppm) were tested, with Ciprofloxacin (CIP) serving as a positive control (Figure 6 and Figure 7 and Table 3). At a concentration of 200 ppm, ZnO NPs exhibited substantial antimicrobial activity across all tested bacterial strains, with ZOI ranging from 18 to 26 mm, demonstrating high efficacy, particularly against Escherichia coli, Staphylococcus aureus, and Bacillus subtilis. At 100 ppm, the ZOIs decreased, ranging from 12 to 19 mm, showing reduced antimicrobial effectiveness. By 50 ppm, the zones were further diminished, ranging from 8 to 16 mm, indicating lower activity. At the lowest concentration of 20 ppm, no significant inhibition was observed for most bacterial strains, highlighting the need for higher concentrations to achieve effective antimicrobial action.

In Gram-negative bacteria such as *Escherichia coli*, which have a complex outer membrane structure, the small size and high surface area of ZnO NPs enable penetration through this barrier effectively (Figure 6). At 200 ppm, *E. coli* exhibited the highest sensitivity with a ZOI of 26 ± 0.2 mm. This effectiveness is due to ZnO NPs generating reactive oxygen species (ROS) like superoxide radicals and hydrogen peroxide, which cause oxidative stress and damage to essential cellular components [46]. Similarly, *Salmonella typhimurium* , and *Klebsiella pneumoniae* showed ZOIs of 20 ± 1.1 mm and 18 ± 0.5 mm at 200 ppm, respectively, reflecting the nanoparticles’ ability to disrupt bacterial membranes and release Zn^2^⁺ ions that interfere with metabolic functions [27]. The reduction in activity at lower concentrations emphasizes the need for higher doses for significant inhibition.

For Gram-positive bacteria, which possess a thick peptidoglycan layer, ZnO NPs displayed considerable activity (Figure 7). At 200 ppm, *Staphylococcus aureus* and *Bacillus subtilis* showed ZOIs of 25 ± 0.2 mm and 25 ± 1.2 mm, respectively, while *Staphylococcus hominis* had a ZOI of 23 ± 0.0 mm. ZnO NPs disrupt the peptidoglycan layer and release Zn^2^⁺ ions that interfere with enzymatic functions [47]. Ciprofloxacin, as a positive control, demonstrated ZOI ranging from 24 mm to 34 mm, indicating competitive antibacterial activity. The MIC values for ZnO NPs are 50 µg/mL against *Escherichia coli*, *Klebsiella pneumoniae*, *Staphylococcus aureus*, and *Staphylococcus hominis*; 100 µg/mL against *Salmonella typhimurium*; 50 µg/mL against *Bacillus subtilis*; and 20 µg/mL against *Candida albicans*. The negative control, sterile deionized water, showed no inhibition, confirming the specific antimicrobial effect of ZnO NPs [48].

### 3.8. Antifungal Activity of Hexagonal ZnO NPs

**Figure 8:**
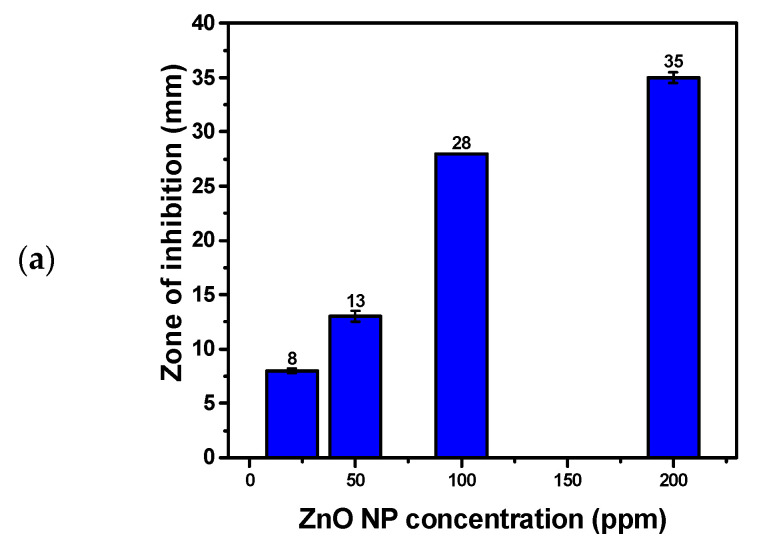

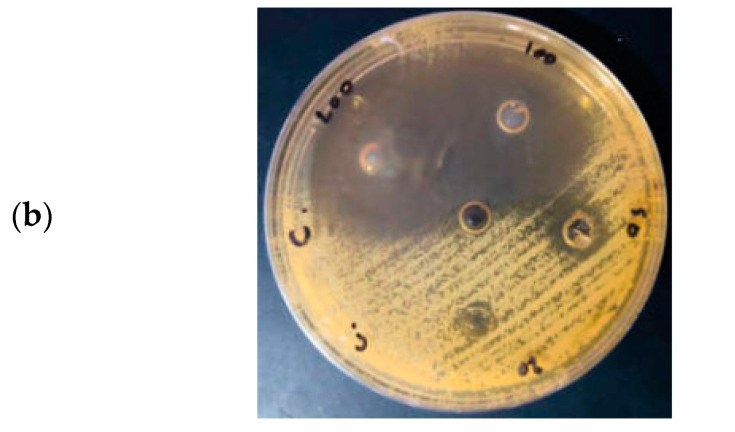
Evaluation of Antifungal Activity of Hexagonal Wurtzite ZnO NPs against *Candida albicans.* (**a**) Bar chart illustrating the ZOI observed for various concentrations of ZnO NPs. (**b**) Camera image of *Candida albicans* culture showing the ZOI around the ZnO NP-treated areas.

ZnO NPs demonstrated notable antifungal activity against *Candida albicans* ATCC 10231, with a ZOI of 35 mm at a concentration of 200 ppm (Figure 8 and Table 4). This significant antifungal effect highlights the broad-spectrum potential of ZnO NPs, extending their application to fungal infections. The underlying mechanism of this activity involves the interaction of ZnO NPs with the fungal cell structure. ZnO NPs disrupt the integrity of the fungal cell membrane, which is crucial for maintaining cellular function and integrity. By penetrating the cell wall and membrane, the nanoparticles induce oxidative stress through the generation of ROS. These ROS cause extensive damage to the cell membrane, leading to leakage of intracellular contents and compromising the cell’s structural integrity. Additionally, the oxidative stress impairs critical cellular components, including DNA, which results in cell death. The incorporation of clove (*Syzygium aromaticum*) and *Thymus capitatus* extracts during ZnO NP synthesis introduces bioactive compounds such as eugenol and thymol, which enhance the antimicrobial efficacy. These compounds not only augment the disruptive effect on the fungal cell membrane but also stabilize the ZnO NPs, improving their penetration and effectiveness. This synergistic effect makes ZnO NPs highly effective against fungal pathogens by targeting and compromising essential aspects of cell structure and function.

### 3.9. Evaluation of Previous Studies

Table 4 compares various green synthesis approaches for ZnO NPs using different plant sources and zinc salts, highlighting their morphologies, sizes, drying conditions, and biological activities. Clove and *Thymus capitatus* extracts yield hexagonal ZnO NPs with a size of 160 nm, which, when dried at 80 °C without high-temperature annealing, exhibit exceptional antioxidant activity (95.2%) and strong antibacterial properties against both Gram-positive and Gram-negative bacteria. In contrast, other studies report diverse morphologies such as prismatic/flower-shaped (Sunflower husk), semi-spherical (*Myrtus communis* L.), and mound-like (Alhagi plant), with varying sizes and biological applications including phytoremediation, eco-friendly pest control, and dye degradation. The synthesis conditions, including drying temperatures and annealing processes, play a crucial role in defining the nanoparticle characteristics and their corresponding biological functions.

## 4. Conclusions

This study introduces a novel green synthesis method for producing ZnO nanoparticles (NPs) using hydrodistilled plant extracts from clove (*Syzygium aromaticum*) and *Thymus capitatus*. Utilizing hydrodistillation avoids the need for organic solvents and high-temperature annealing, enabling the precipitation of ZnO NPs at room temperature. The synthesized ZnO NPs, with sizes around 160 nm and a well-defined hexagonal wurtzite structure, have a crystallite size of approximately 30 nm as determined by X-ray diffraction (XRD). Energy-dispersive X-ray spectroscopy (EDX) confirms the elemental composition of the ZnO NPs, showing high carbon content (63.9 wt.%), indicative of the phytochemicals from the plant extracts loaded onto the ZnO NPs surface. Ultraviolet-visible (UV–Vis) spectroscopy reveals a prominent absorption peak at 370 nm and a bandgap of approximately 2.8 eV. This reduction in bandgap is attributed to lattice defects or the adsorption of phytochemicals, which introduce additional electronic states within the bandgap. The ZnO NPs demonstrated exceptional antioxidant activity with a DPPH radical scavenging rate of 95.2%, surpassing that of ascorbic acid at the same concentration. The NPs also showed significant antibacterial properties with inhibition zones against *Escherichia coli* ATCC 25922, *Salmonella typhimurium* ATCC 14028, *Klebsiella pneumoniae* ATCC 13883, *Staphylococcus aureus* ATCC 25932, *Staphylococcus hominis* ATCC 27844, *Bacillus subtilis* ATCC 25973, and *Candida albicans* ATCC 10231, comparable to or exceeding those of ciprofloxacin at 200 ppm. These findings highlight the potential of the synthesized ZnO NPs as broad-spectrum antimicrobial agents and indicate the benefits of using green synthesis methods. This approach provides an environmentally friendly, cost-effective means of producing ZnO NPs with enhanced biological activities and applications in pharmaceuticals, food preservation, and novel antimicrobial products.

## Figures and Tables

**Table 1:** Antioxidant and Antibacterial Activity of Synthesized ZnO NPs Using Combined Plant Extracts of clove and *Thymus Capitatus. Standard deviation is based on three measurements.*

Exp. No.	Synthesis Conditions			Characteristics		
	ZnCl_2_ (g)	*Clove* Extract (mL)	*Thymus* *Capitatus* Extract (mL)	Antioxidant (% DPPH Inhibition)	*Gram-Negative* *K. pneumoniae* (ZOI mm)	*Gram-Positive* *B. subtilis* (ZOI mm)
1	3.0	15	5	85.5	14 ± 0.8	10 ± 0.5
2	3.0	5	15	86.3	12 ± 0.5	15 ± 0.8
3	3.0	10	10	91.2	15 ± 0.5	16 ± 1.1
4	4.0	10	10	92.0	14 ± 1.0	15 ± 0.5
5	5.5	10	10	93.4	11 ± 0.75	12 ± 0.8
6	2.0	10	10	95.2	18 ± 0.5	25 ± 0.2
7	4.0	5	5	93.3	9 ± 0.2	10 ± 0.8

Note: The Ciprofloxacin (CIP) Disk Diffusion Test (5 µg per disk) shows a ZOI of 34 ± 0.8 mm for Klebsiella pneumoniae and 24 mm for Bacillus subtilis. Ascorbic acid demonstrated a DPPH Inhibition of 89%.

**Table 2:** Comparative analysis of antioxidant activity of hexagonal ZnO NPs and *Ascorbic Acid* (reference antioxidant) as determined by DPPH radical scavenging assay at various ZnO NP concentrations.

Sample Concentration	ZnO NPs		Ascorbic Acid	
	Absorbance (a.u.) ± SD	DPPH Inhibition (%)	Absorbance (a.u.) ± SD	DPPH Inhibition (%)
220 µ/mL	0.70 ± 0.01	30.0%	0.69 ± 0.05	31.0%
500 µ/mL	0.437 ± 0.05	56.3%	0.45 ± 0.02	55.0%
750 µ/mL	0.177 ± 0.02	82.3%	0.19 ± 0.05	81.0%
1000 µ/mL	0.048 ± 0.01	95.2%	0.11 ± 0.05	89.0%
IC50	/	434.06 µg/mL	/	442.96 µg/mL

**Table 3:** Antimicrobial Activity and Minimum Inhibitory Concentration (MIC) of ZnO Nanoparticle Dispersion Against Various Microbial Strains, Including Gram-Positive Bacteria, Gram-Negative Bacteria, and Yeast.

Microbial Strains Used	ZnO NP Dispersion				CIP (Positive Control)	Deionized Water (Negative Control)	MIC (µg/mL)
	200 ppm	100 ppm	50 ppm	20 ppm	5 µg/Discs		
*Escherichia coli* ATCC 25922	26 ± 0.2	19 ± 1.1	10 ± 0.3	Nill	34 ± 0.2	Nill	50
*Salmonella typhimurium* ATCC 14028	20 ± 1.1	15 ± 0.0	Nill	Nill	31 ± 0.5	Nill	100
*Klebsiella pneumoniae* ATCC 13883	18 ± 0.5	16 ± 0.7	10.5 ± 0.5	Nill	34 ± 1.0	Nill	50
*Staphylococcus aureus* ATCC 25932	25 ± 0.2	15 ± 1.0	10 ± 0.8	Nill	30 ± 0.8	Nill	50
*Staphylococcus hominis* ATCC 27844	23 ± 0.0	14 ± 0.5	8 ± 0.0	Nill	29 ± 0.4	Nill	50
*Bacillus subtilis* ATCC 25973	25 ± 1.2	12 ± 0.8	9 ± 0.2	Nill	24 ± 0.0	Nill	50
*Candida albicans* ATCC 10231	35 ± 0.5	28 ± 0.0	13 ± 0.5	8 ± 0.2	/	Nill	20

**Table 4:** Evaluation of previous studies.

Plant Source	Zn Salt	Shape and Size (nm)	Drying and Annealing Conditions	Biological Properties	Ref.
Non	ZnCl_2_	20–350 nm Semi-spherical	100	Not tested	[36]
*Sunflower husk*	Zn nitrate	prismatic/flower shaped	Dried in a vacuum oven at 60 °C overnight	Phytoremediation of toxic chemicals from water bodies	[49]
*Myrtus communis*	Zn acetate	semi-spherical, 55 nm	dried at 60 °C overnight	Eco-friendly alternative to chemical insecticides	[50]
*Plectranthus amboinicus*	Zn sulphate	hexagonal close-packed lattice	Air dried	90% antibacterial effectiveness against *S. aureus* and *E. coli*; 85.29% degradation of Safranin-O dye, 81.57% of Malachite green dye	[51]
*Alhagi*	Zn nitrate	mounds-like, 55 nm	dried at 60 °C overnight	No studies	[52]
*Pineapple peel*	zinc nitrate	flower-like	dried in an oven at 80 °C overnight	fascinating optical properties	[53]
*Raphanus sativus*	zinc acetate	Hexagonal wurtzite, 66 nm	dried at 50 °C	Anticancer and antibacterial, causing ROS generation and activating apoptotic pathways	[54]
*Clove* and *Thymus capitatus*	Zn chloride	Hexagonal, 160 nm	Dried at 80 °C and not annealed at high temperature	95.2% antioxidant activity, potent antibacterial effects against both Gram-(+) and Gram-(−) bacteria	This work

## Data Availability

The original contributions presented in the study are included in the article, further inquiries can be directed to the corresponding author.
